# Prevalence, Potential Virulence, and Genetic Diversity of *Listeria monocytogenes* Isolates From Edible Mushrooms in Chinese Markets

**DOI:** 10.3389/fmicb.2018.01711

**Published:** 2018-07-27

**Authors:** Moutong Chen, Jianheng Cheng, Qingping Wu, Jumei Zhang, Yuetao Chen, Haiyan Zeng, Qinghua Ye, Shi Wu, Shuzhen Cai, Juan Wang, Yu Ding

**Affiliations:** ^1^State Key Laboratory of Applied Microbiology Southern China, Guangdong Provincial Key Laboratory of Microbial Culture Collection and Application, Guangdong Open Laboratory of Applied Microbiology, Guangdong Institute of Microbiology, Guangzhou, China; ^2^College of Food Science, South China Agricultural University, Guangzhou, China; ^3^Department of Food Science and Technology, Jinan University, Guangzhou, China

**Keywords:** *Listeria monocytogenes*, edible mushrooms, antibiotic resistance profiles, multilocus sequence typing, virulence profiles, serotypes

## Abstract

*Listeria monocytogenes*, an intracellular foodborne pathogen, is capable of causing listeriosis, such as meningitis, meningoencephalitis, and abortion. In recent years, the occurrence of *Listeria monocytogenes* in edible mushroom products has been reported in several countries. There are no guidelines for qualitative and quantitative detection of *L. monocytogenes* in mushroom products in China. Therefore, this study aimed to investigate the prevalence and contamination level of *L. monocytogenes* in edible mushrooms in Chinese markets and to determine the antibiotic resistance and sequence types (STs) of these isolates to provide data for risk assessments. Approximately 21.20% (141/665) of edible mushroom samples were positive for *L. monocytogenes*, while 57.44% (81/141) of positive samples contained contamination levels of less than 10 MPN/g. The 180 isolates derived from positive samples belonged to serogroup I.1 (1/2a-3a, *n* = 111), followed by serogroup II.2 (1/2b-3b-7, *n* = 66), and serogroup III (4a-4c, *n* = 3). Antibiotic susceptibility testing showed that over 95% of *L. monocytogenes* isolates were resistant to penicillin, ampicillin, oxacillin, and clindamycin, while over 90% were susceptible to 16 antibiotic agents, the mechanisms of resistance remain to be elucidated. According to multilocus sequencing typing, the 180 isolates represented 21 STs, one of which was identified for the first time. Interestingly, ST8 and ST87 were predominant in edible mushroom products, indicating that specific STs may have distinct ecological niches. Potential virulence profiles showed that most of the isolates contained full-length *inlA* genes, with novel premature stop codons found in isolate 2035-1LM (position 1380, TGG→TGA) and 3419-1LM (position 1474, CAG→TAG). Five isolates belonging to serogroup II.2 carried the *llsX* gene from *Listeria* pathogenicity island (LIPI)-3, present in ST224, ST3, and ST619; 53 (29.44%) harbored the *ptsA* gene from LIPI-4, presenting in ST3, ST5, ST87, ST310, ST1166, and ST619. Five potential hypervirulent isolates carrying all three of these virulence factors were identified, suggesting edible mushrooms may serve as possible transmission routes of potential hypervirulent *L. monocytogenes*, which may be of great public health concern to consumers. Based on our findings, the exploration of novel approaches to control *L. monocytogenes* contamination is necessary to ensure the microbiological safety of edible mushroom products.

## Introduction

*Listeria monocytogenes* is an important intracellular foodborne pathogen that causes listeriosis, a serious disease associated with meningitis, meningoencephalitis, and abortion, especially in immunocompromised individuals, pregnant women, the elderly, and neonates. It remains a major public health concern due to its high mortality rate of up to 20–30% (Lomonaco et al., 2015). *Listeria monocytogenes* is capable of surviving in various adverse environments, such as low temperature, high salinity, and a wide range of pH values (Gandhi and Chikindas, 2007). In addition, *L. monocytogenes* biofilm exhibits a strong ability to resist disinfectants, resulting in persistent contamination of food plants (nakamura et al., 2013; Piercey et al., 2017).

*Listeria monocytogenes* can be divided into 13 serotypes based on the somatic(O) and flagellar (H) antigens. Doumith et al. (2004) further divided the 13 serotypes into five serogroups, designated as I.1 (containing serotypes 1/2a-3a), I.2 (1/2c-3c), II.1 (4b-4d-4e), II.2 (1/2b-3b-7), and III (4a-4c). Previous studies demonstrated differences in virulence and ecological distribution among different *L. monocytogenes* serotypes. Serotypes 1/2a, 1/2b, and 4b are dominant among clinical *L. monocytogenes* isolates derived from patients with listeriosis (Cartwright et al., 2013). The ability of *L. monocytogenes* to invade and proliferate inside host cells relies on a series of virulence factors, mainly distributed in *Listeria* pathogenicity islands 1(LIPI-1) and 2 (LIPI-2). LIPI-1 harbors several important genes, including *prfA, plcA, hly, mpl, actA*, and *plcB*, that participate in host invasion and cellular proliferation. LIPI-2, a 22 kb gene cluster composed of many internalin genes in *L. ivanovii* (Vázquez-Boland et al., 2001). In *L. monocytogenes*, internalin A (*inlA*) and internalin B (*inlB*) genes mainly mediate the ability of *L. monocytogenes* to invade and cross diverse cellular barriers during the infection process. Previous studies have demonstrated that both LIPI-1 and LIPI-2 are widely distributed in each *L. monocytogenes* serotype; thus, it is not possible to evaluate the potential pathogenicity of *L. monocytogenes* strains simply by detecting the presence of LIPI-1 and LIPI-2 (Vázquez-Boland et al., 2001). In recent years, two novel virulence factors were discovered in some lineages or clonal complexes (CC) of *L. monocytogenes*, demonstrating the heterogeneity of this pathogen in terms of virulence. Listeriolysin S (LLS), designated as *Listeria* pathogenicity island 3 (LIPI-3), can enhance the hemolytic and cytotoxic activity of *L. monocytogenes* and is only found in some lineage I strains (Cotter et al., 2008). In 2016, Maury et al. reported a newly identified pathogenic cluster, known as *Listeria* pathogenicity island 4 (LIPI-4), which mainly includes the cellobiose-family phosphotransferase system (PTS). This CC4-associated PTS is specifically involved in the selective tropism of *L. monocytogenes* for the central nervous system (CNS) and the fetal-placental unit with high clinical relevance (Maury et al., 2016). These findings demonstrate the ability of *L. monocytogenes* to vary in regard to pathogenicity at the intraspecies level.

Edible mushrooms are popular worldwide owing to their taste and nutritional value. According to the Food and Agriculture Organization reports, mushroom production has reached approximately 40 million tons worldwide. China is the largest mushroom producer in the world, with a total output of up to 31.7 million tons in 2013, accounting for 75% of the global mushroom production (Zhang et al., 2015). *Lentinula edodes, Auricularia auricula-judae, Pleurotus ostreatus, Flammulina velutipes*, and *Agaricus bisporus* are the main mushroom species used for production, accounting for 9.0, 6.8, 5.4, 2.7, and 3.4 million tons, respectively, in 2016 (China Edible Fungi Association, 2017). In recent years, the occurrence of *Listeria monocytogenes* in edible mushroom products has been reported in several countries (Cordano and Jacquet, 2009; Venturini et al., 2011; Viswanath et al., 2013; Wu et al., 2015). Canada and the United States now have standards and policies designed to minimize the potential risk of foodborne listeriosis. However, there are no corresponding guidelines for the qualitative and quantitative detection of *L. monocytogenes* in mushroom products in China. Our previous study showed that 31.5% of edible mushroom samples were positive for *L. monocytogenes* in South China, particularly *F. velutipes* products (Chen et al., 2015). We have also reported that the surfaces of mycelium-scraping machinery may serve as the primary contamination source of *L. monocytogenes* in the *F. velutipes* production process (Chen et al., 2014a). Edible mushrooms, therefore, may be a potential transmission vehicle of *L. monocytogenes* infection for consumers. It is thus necessary to survey the occurrence and characteristics of *L. monocytogenes* isolated from mushroom products to provide basic data for risk assessment and formulating standards of food microbiology.

The objective of this study was to: (i) evaluate the contamination levels of *L. monocytogenes* in edible mushroom samples from Chinese retail food systems, both qualitatively and quantitatively, and (ii) determine the phenotypic and genotypic characteristics of *L. monocytogenes* for risk analysis of edible mushroom production systems.

## Materials and Methods

### Sample Collection

From July 2012 to April 2016, a total of 665 (237 packages from the processing facilities and 432 loose samples) retail edible mushroom samples were collected from rural markets, open-air markets, and large supermarkets in China. Samples comprised *F. velutipes* (*n* = 209), *P. ostreatu*s (*n* = 104), *Lentinula edode*s (*n* = 104), *Pleurotus eryngii* (n = 91), *Hypsizygus marmoreus* (*n* = 79), *Volvariella volvacea* (*n* = 17), *Pleurotus geesteranus* (*n* = 12), and other mushrooms (*n* = 49), including *Agaricus bisporus, Pleurotus nebrodensis, Auricularia auricula, Coprinus comatus, Cordyceps militaris, Chanterellus* sp., and *Agrocybe cylindracea*. Samples were placed in insulated shipping coolers with frozen gel packs placed on the sides, between samples, and on top of the samples. All samples were kept below 4°C during the transportation process, and tests were initiated within 4 h of receiving them.

### Qualitative and Quantitative Analysis

According to the National Food Safety Standard of China (GB4789.30-2010, National Standard of the People’s Republic of China, 2010), an enrichment method with minor modifications was used for qualitative detection (Chen et al., 2014b). In brief, samples were analyzed for the presence of *L. monocytogenes* by homogenizing 25 g of each sample in 225 mL *Listeria* enrichment broth 1 (LB1; Guangdong Huankai Co. Ltd., Guangzhou, China). Homogenates were incubated at 30°C for 24 h; thereafter 0.1 mL of the *Listeria* enrichment broth 1 (LB1) enrichment culture was transferred to 10 mL LB2 at 30°C for 24 h. A loop (about 10 μL) of the LB2 enrichment culture was streaked onto *Listeria* selective agar plates (Guangdong Huankai Co. Ltd.) and incubated at 37°C for 48 h. Three to five presumptive colonies that were typically blue in color with a white halo were selected for identification of *L. monocytogenes* using the Microgen ID *Listeria* identification system (Microgen, Camberley, United Kingdom) according to the manufacturer’s instructions.

For the determination of the most possible number (MPN), the workflow was adapted from a previous study by Gombas et al. (2003). Briefly, a 9-tube MPN method was used. The nine tubes were divided into three sets of three tubes. The second and third sets of tubes contained 10 mL of Fraser broth medium. Three aliquots (10, 1, and 0.1 mL) of the sample homogenate were dispensed into the three sets, representing 1.0, 0.1, and 0.01 g of the original sample, respectively. The tubes were incubated at 30 ± 2°C for 24 ± 2 h, and 0.1 mL from each tube was transferred to a new tube containing 10 mL of Fraser broth. The tubes were incubated at 30 ± 2°C for 26 ± 2 h. Darkened Fraser tubes were subjected to assessment. If a Fraser broth tube did not darken, it was examined again after an additional 26 ± 2 h of incubation. The MPN value was determined on the basis of the number of positive tubes in each of the three sets using an MPN table (Chen et al., 2014b).

### Serogroup Analysis

Genomic DNA was extracted from *L. monocytogenes* using a Bacterial Genomic DNA Purification Kit (Dongsheng Biotech. Inc., Guangzhou, China) according to the manufacturer’s instructions. The serogroup analysis of 180 isolates (Supplementary Table S1) was performed using a multiplex PCR according to the methods of Doumith et al. (2004). The 13 serotypes of *L. monocytogenes* were classified into five distinct serogroups, that is, I.1 (1/2a-3a), I.2 (1/2c-3c), II.1 (4b-4d-4e), II.2 (1/2b-3b-7), and III (4a-4c). Primers are shown in Supplementary Table S2. The PCR was performed with an initial denaturation step at 94°C for 3 min; 35 cycles at 94°C for 35 s, 53°C for 50 s, and 72°C for 60 s; and a final extension at 72°C for 7 min in a thermocycler (Applied Biosystems, Carlsbad, CA, United States). Eight microliters of amplicon were separated on a 2% agarose gel in TAE buffer. The PCR products were visualized with Goldview^®^ staining (0.005%, v/v).

### Antimicrobial Susceptibility Testing

Since no resistance criteria exist for *Listeria* antibacterial susceptibility testing in the Clinical and Laboratory Standards Institute guidelines (Clinical and Laboratory Standards Institute, 2014) for tested AMs, the criteria for staphylococci were applied. A total of 22 antibiotic agents were tested at specific concentrations per disk: ampicillin (10 μg), cephalothin (30 μg), chloramphenicol (30 μg), ciprofloxacin (5 μg), erythromycin (15 μg), gentamicin (10 μg), kanamycin (30 μg), rifampin (5 μg), doxycycline (30 μg), levofloxacin (5 μg), penicillin (10 U), tetracycline (30 μg), vancomycin (30 μg), sulfamethoxazole with trimethoprim (23.75/1.25 μg), sulbactam/ampicillin (10/10 μg), meropenem (10 μg), clindamycin (2 μg), linezolid (30 μg), amoxicillin/clavulanic acid (10 μg), oxacillin (1 μg), ofloxacin (5 μg), and streptomycin (10 μg) (Oxoid, Basingstoke, United Kingdom). Antimicrobial susceptibility tests were performed according to the methods of a previous study (Kovacevic et al., 2012). *Staphylococcus aureus* ATCC 25923 and *Escherichia coli* ATCC 25922 were used as quality control strains. Zones of inhibition were measured with a precision caliper to the nearest 0.01 mm. Isolates exhibiting resistance to at least three classes of tested antimicrobial agents were considered to be multidrug-resistant strains (Magiorakos et al., 2012).

### Identification of Potential Hypervirulent Isolates

LIPI-3 and LIPI-4 genes (*llsX* and *ptsA*, respectively) were amplified by PCR to identify potential hypervirulent *L. monocytogenes* isolates (Clayton et al., 2011; Maury et al., 2016). The presence of premature stop codons (PMSCs) in the *inlA* gene was determined by amplicon sequencing. The full-length *inlA* gene (2403 bp) was amplified in 180 isolates using external primers, and internal primers were used for sequencing (Supplementary Table S2; Wu et al., 2016). The *inlA* sequences were assembled using DNAMAN software (version 8). By comparing the obtained *inlA* sequence data to that of the *L. monocytogenes* EGDe reference strain, sites of PMSC mutations in *inlA* were determined (Van Stelten et al., 2010; Gelbicova et al., 2015).

### Multilocus Sequence Typing (MLST) Analysis

The MLST scheme used in this study was previously reported by Ragon et al. (2008) (Supplementary Table S3). Briefly, each 50 μL PCR reaction contained 5.0 μL 10× PCR buffer (Takara, Dalian, China), 1.5 mM MgCl_2_, 0.2 mM of each dNTP, 0.4 mM of each primer, 1.25 U Taq polymerase, and 1 μL genomic DNA. The PCR was performed using the following program: initial denaturation at 94°C for 3 min; 35 cycles at 94°C for 30 s, 52°C (45°C for *bglA)* for 1 min, and 72°C for 2 min; followed by a final elongation at 72°C for 10 min. The PCR products were purified and sequenced by Invitrogen (Carlsbad, CA, United States). Allele numbers were assigned according to variations in housekeeping genes, multilocus sequence types (STs), and CCs via the *Listeria* MLST database at the Pasteur Institute website^1^. The seven concatenated housekeeping genes were aligned, and a neighbor-joining tree was constructed to analyze the phylogenetic relationships among different STs using Mega 7.0 software (Kumar et al., 2016). Simpson’s index of discrimination (DI) was calculated to determine the discriminatory power of the MLST method according to a previous study by Hunter and Gaston (1988).

## Results

### Prevalence and Quantification of *L. monocytogenes* in Edible Mushroom Samples

**FIGURE 1:**
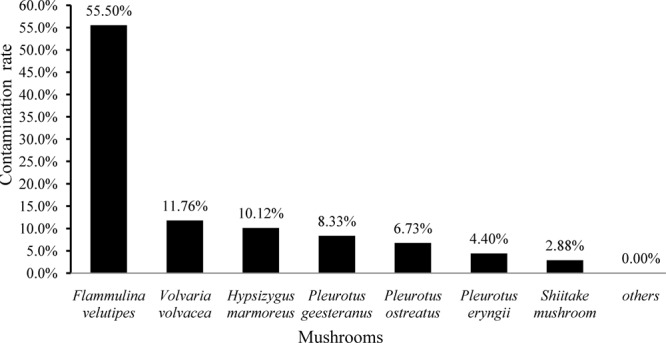
Prevalence of *Listeria monocytogenes* among different edible mushroom species.

In the present study, 141 (21.20%) samples were positive for *L. monocytogenes* out of 665 edible mushrooms samples collected from Chinese markets. As shown in **Figure 1**, the contamination rate of *L. monocytogenes* was 55.50% (116/209) in *F. velutipes* samples, 11.76% in *V. volvacea*, 10.12% in *H. marmoreus*, 6.73% in *P. ostreatus*, 4.40% in *P. eryngii*, and 2.88% in *L. edodes*. All other mushroom samples were negative for *L. monocytogenes*. For quantitative analysis, among 141 positive samples, the contamination level of 43 samples was over 110 MPN/g, while that of 81 samples was between 0.3 and 10 MPN/g. Contamination levels also varied among the different mushroom species. Among 116 positive *F. velutipes* products, 42 (36.2%) samples exceeded 110 MPN/g, while 57 (49.14%) exhibited contamination levels between 0.3 and 10 MPN/g. In contrast, only one *P. ostreatus* sample exhibited contamination of over 100 MPN/g, with a total of 24 positive samples ranging from 0.3 to 10 MPN/g in other mushroom products (**Table 1**).

**Table 1:** The contamination levels of *Listeria monocytogenes* in different edible mushrooms.

Samples	0.3 ≤ MPN<10	10 ≤ MPN<110	≥110	Total
*Flammulina velutipes*	57	17	42	116
*Pleurotus ostreatus*	6	0	1	7
*Lentinula edodes*	3	0	0	3
*Pleurotus eryngii*	4	0	0	4
*Hypsizygus marmoreus*	8	0	0	8
*Volvariella volvacea*	2	0	0	2
*Pleurotus geesteranus*	1	0	0	1
Others	0	0	0	0
Total	81	17	43	141

### Serogroup Analysis

**FIGURE 2:**
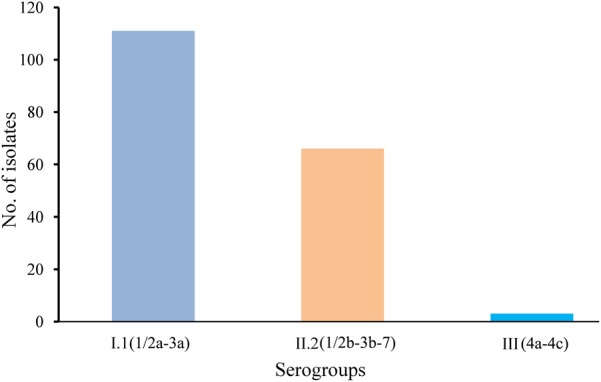
Serogroup distributions of *Listeria monocytogenes* derived from retail edible mushroom samples.

A total of 180 isolates collected from 141 *L. monocytogenes*-positive samples were submitted for serogroup analysis. As shown in **Figure 2**, serogroup I.1 (1/2a-3a) and II.2 (1/2b-3b-7) were dominant among edible mushrooms, with 61.67% (111/180) of the *L. monocytogenes* isolates belonging to serogroup I.1 and 36.67% (66/180) belonging to serogroup II.2. Three isolates were classified as serogroup III (4a-4c), all of which were isolated from *F. velutipes* samples. Interestingly, serogroups I.2 (1/2c-3c) and II.1 (4b-4d-4e) were not identified in edible mushroom samples.

### Antibiotic Susceptibility

**FIGURE 3:**
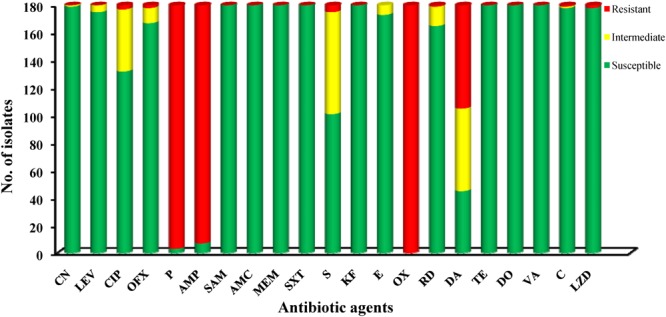
Antibiotic susceptibilities of *Listeria monocytogenes* isolates.

A panel of 22 antibiotic agents was used for antimicrobial resistance analysis of 180 isolates. According to the breakpoint criteria for *S. aureus* or *Enterococcus* spp., oxacillin (100%), penicillin (98.33%), and ampicillin (96.11%) were the top three antibiotics for which *L. monocytogenes* isolates exhibited resistance. In contrast, all isolates were susceptible to eight antibiotic agents, including sulbactam/ampicillin, amoxicillin/clavulanic acid, meropenem, sulfamethoxazole with trimethoprim, cephalothin, tetracycline, doxycycline, and vancomycin. In addition to these eight agents, over 90.0% of isolates were susceptible to eight additional antibiotics, namely kanamycin, gentamycin, levofloxacin, ofloxacin, erythromycin, rifampin, chloramphenicol, and linezolid. In consideration of the fact that a moderately resistant isolate may become a resistant strain under certain circumstances (Ruiz-Bolivar et al., 2011), all 180 isolates were classified into 35 antibacterial resistance profiles, highlighting the existence of 97 multidrug-resistant isolates. Notably, 7.22% (13/180) of isolates were resistant to up to five classes of antibiotic agents (**Figure 3**).

### Identification of Hypervirulent Isolates

In this study, the presences of the *llsX* and *ptsA* genes were detected by PCR. A total of 53 (29.44%) isolates harbored *ptsA*, including 44 (97.78%) isolates belonging to ST87, three (100%) in ST3, two in ST1166, one in ST5, ST224, ST310, and ST619, respectively. None of the isolates belonging to serogroup I and III harbored *ptsA*. Only five (2.78%) isolates (2919-1LM, 3949-1LM, 2921-1LM, 1520-1LM, and 3950-1LM) carried the *llsX* and *ptsA*, all of which belonged to serogroup II.2. The presence of PMSCs in the *inlA* gene was determined in all 180 isolates by DNA sequencing. According to the full-length sequence of *inlA* in the reference *L. monocytogenes* EGDe, 98.89% of isolates carried full-length *inlA* genes. Two isolates (2035-1LM and 3419LM) were found to carry PMSCs in *inlA*. In the isolate 2035-1LM, sequence analysis revealed a nonsense mutation at position 1474, which resulted in changing a glutamine codon to a stop codon (CAG→TAG). In 3419-1LM, a nonsense mutation at position 1380 changed a tryptophan codon to a stop codon (TGG→TGA). According to previously known PMSC mutation types (Gelbicova et al., 2015), both of these point mutations in the *inlA* gene are novel.

### Multilocus Sequence Typing Analysis

**FIGURE 4:**
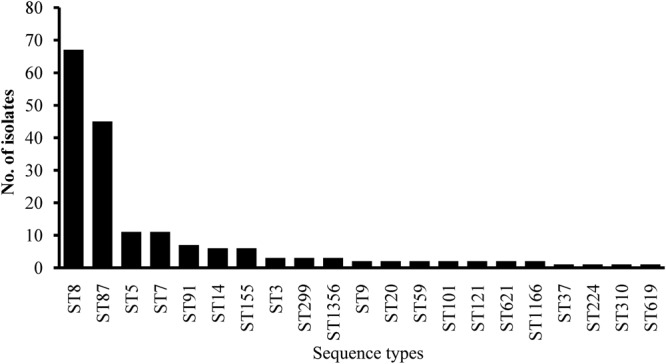
Sequence type distributions of *Listeria monocytogenes* isolates.

**FIGURE 5:**
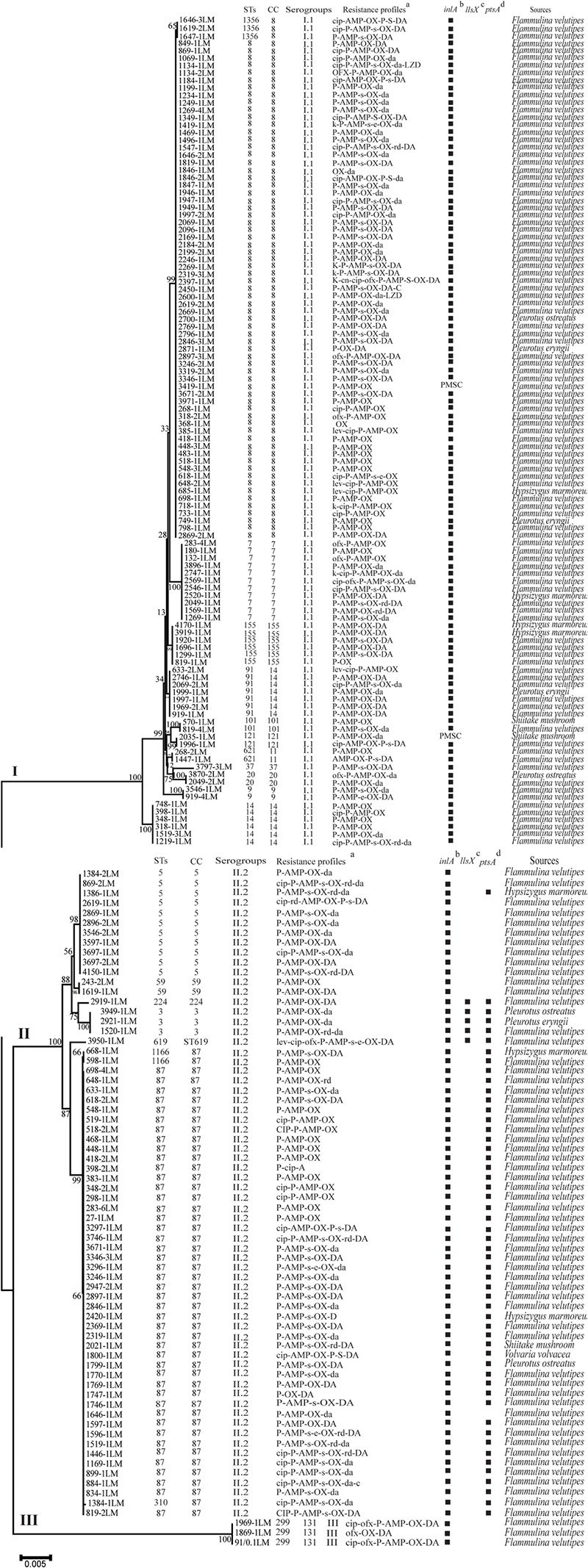
The characteristics of *Listeria monocytogenes* isolates recovered from retail edible mushrooms. (a) T(t), tetracycline; P(p), penicillin; CIP(cip), ciprofloxacin; K(k), kanamycin; A(a), ampicillin; C(c), cephalothin; CN(cn), gentamycin; E(e), erythromycin; D(d), doxycycline; SXT(sxt), sulfamethoxazole with trimethoprim; V(v), vancomycin; R(r), rifampin; L(l), levofloxacin; ND, not detected. A slash (/) indicates no resistance. Antibiotic abbreviations in uppercase indicate resistance, while those in lowercase indicate intermediate resistance. (b) PMSC, premature stop codons in *inlA*; squares indicate the presence of full-length *inlA*. (c) Squares indicate the presence of *llsX*. (d) Squares indicate the presence of *ptsA*. The sequences were aligned by MAFFT (version 7.273) and a neighbor-joining tree based on MLST of seven housekeeping genes was established by MEGA 7.0 with 1000 replications in the bootstrap test. Bootstrap values are shown at the nodes.

Multilocus Sequence Typing was used for typing of *L. monocytogenes* isolates. All 180 isolates were grouped into 21 STs belonging to 18 CCs with a DI of 0.79 (**Figure 4**). A good correlation between STs and serogroups was observed, with serogroups I.1, II.2, and III divided into three clusters (designated as I, II, and III) based on the MLST of seven housekeeping genes (**Figure 5**). All 111 isolates belonging to serogroup I.1 were included in Cluster I, including 12 STs (ST14, ST9, ST20, ST621, ST121, ST101, ST91, ST155, ST37, ST7, ST1356, and ST8). Eight STs were grouped into Cluster II, including ST5, ST59, ST224, ST3, ST619, ST1166, ST310, and ST87 (**Figure 4**). Three isolates (1969-1LM, 1869-1LM, and 91/0.1LM) classified as serogroup III were grouped into Cluster III. In addition, three isolates (1646-3LM, 1619-2, and 1647-1LM) were found to belong to the novel ST1356. ST8/CC8 (serogroup I.1) and ST87/CC87 (serogroup II.2) accounted for 37.22 and 25.00% of *L. monocytogenes* isolates, respectively, derived from edible mushroom products, indicating that ST8 and ST87 were predominant among edible mushroom products.

## Discussion

*Listeria monocytogenes* is an important foodborne pathogen worldwide, causing serious cases of listeriosis following the consumption of contaminated foods. Edible mushrooms are popular worldwide, especially in Asia. Some developed countries have formulated policies and food microbiological safety standards to minimize the consumption of contaminated edible mushrooms. Therefore, it is of great interest to monitor the prevalence and contamination levels of *L. monocytogenes* in edible mushrooms.

The present study was systematically conducted to determine the occurrence, genetic diversity, and potential risk of *L. monocytogenes* isolates in edible mushrooms in the Chinese food system. Qualitative analysis showed that 21.20% of edible mushroom samples were positive for *L. monocytogenes*, with 57.45% of the positive samples with contamination levels below 10 MPN/g according to quantitative analysis. This prevalence rate of *L. monocytogenes* is similar to that reported for a commercial fresh mushroom processing environment (Murugesan et al., 2015) but is significantly higher than that reported in mushroom products in Seattle (Samadpour et al., 2006), an *Agaricus bisporus* production facility on the campus of Pennsylvania State University in the United States (Viswanath et al., 2013), and other countries as reviewed by Lianou and Sofos (2007). Previous study reported the contamination level of foods that has caused listeriosis outbreaks were mostly ≥10^4^ CFU/g (Miya et al., 2010), the contamination level of *L. monocytogenes* in edible mushroom products was quite low (**Table 1**), suggesting that the edible mushroom products were relatively safe. However, *L. monocytogenes* is ability to grow on the fresh mushrooms during postharvest period, which populations may increase between 1 and 2 log units within the first 48 h (González-Fandos et al., 2001). It is necessary to explore the effective treatments to control *L. monocytogenes* on fresh mushroom products. It is interesting to note that the contamination rate of *L. monocytogenes* was higher in *F. velutipes* samples (55.50%) than that of the different kinds of edible mushroom samples and that 36.21% of positive *F. velutipes* samples exhibited contamination levels exceeding 110 MPN/g. These results demonstrate that *F. velutipes* should be considered a potential transmission source of *L. monocytogenes*. Chen et al. (2014a) reported that the surfaces of mycelium-scraping machinery may be the main source of *L. monocytogenes* contamination in *Flammulina velutipes* plants. In addition, *L. monocytogenes* also contaminated the harvesting room of *Flammulina velutipes* products (Chen et al., 2014a; Murugesan et al., 2015). A thorough sanitation of standard operation protocol for manufacturing machinery and associated environments should be formulated to ensure the microbiological safety of mushroom products. Since *L. monocytogenes* in raw foods may result in cross-contamination of ready-to-eat foods in storage, processing, and the associated environment, this may become a major concern to consumers in China. In addition, the underlying mechanism of the high prevalence of *L. monocytogenes* in *F. velutipes* products should be investigated in future studies.

As foodborne *L. monocytogenes* is the main cause of listeriosis, it is important to monitor the antibiotic resistance profiles of isolates as a reference for listeriosis treatment. *Listeria monocytogenes* may acquire antibiotic resistance via several mechanisms, including conjugative mobilization (Toomey et al., 2009), self-transferable plasmids (Roberts et al., 1996), and efflux pumps (Jiang et al., 2012). In this study, a panel of 22 antibiotic agents was used to test for antibiotic susceptibility of the isolates. In total, 53.89% of isolates exhibited multidrug resistance. Over 90% of isolates were susceptible to 16 antibiotic agents, including sulbactam/ampicillin, amoxycillin/clavulanic acid, meropenem, sulfamethoxazole with trimethoprim, cephalothin, tetracycline, doxycycline, vancomycin, kanamycin, gentamicin, levofloxacin, ofloxacin, erythromycin, rifampin, chloramphenicol, and linezolid, suggesting that these antibiotics are still effective for the treatment of listeriosis. However, most *L. monocytogenes* isolates were resistant to oxacillin, penicillin, ampicillin, and clindamycin, which is consistent with findings from *L. monocytogenes* isolates from foods and food processing environments in China and other countries (Harakeh et al., 2009; Camargo et al., 2015; Lotfollahi et al., 2017; Wang et al., 2017). In contrast, previous studies reported that no penicillin-resistant isolates were found in China (Chao et al., 2007; Yan et al., 2014), indicating that the resistance of *L. monocytogenes* to penicillin may be on the rise in China. Since penicillin and ampicillin are considered first-line therapies for the treatment of listeriosis, surveillance of the variation in the antibiotic resistance of foodborne *L. monocytogenes* is critical. In this study, no correlation was observed between antibiotic resistance and *L. monocytogenes* serogroups or STs. Thus, a comprehensive study is needed to explore the potential molecular mechanisms of antibiotic resistance in *L. monocytogenes*.

Serotypes 1/2a, 1/2b, and 4b are considered hypervirulent isolates among the 13 serotypes of *L. monocytogenes*. In the present study, serogroups I.1 (1/2a-3a) and II.2 (1/2b-3b-7) were most prevalent in edible mushroom products, consistent with results of *L. monocytogenes* isolates derived from raw foods in South China (Chen et al., 2015; Wu et al., 2015). Similarly, serotype (1/2b-3b-7, 30.1%), serotype (1/2a-3a, 40.8%), and serotype (4b-4d-4e, 29.1%) were found in *Agaricus bisporus* mushroom production (Pennone et al., 2018). In contrast, Murugesan et al. (2015) reported that serotype 1/2c was predominant in a commercial fresh mushroom processing environment, and strains belonging to serotype 4a were isolated from a small-scale mushroom (*Agaricus bisporus*) production facility (Viswanath et al., 2013), indicating that specific serotypes of *L. monocytogenes* may favor distinct ecological niches in different mushroom products. *Listeria monocytogenes* isolates belonging to the same serotype and CC may differ in their virulence characteristics, as reviewed by Orsi et al. (2011); thus, it is not possible to assess the virulence of *L. monocytogenes* simply by detecting the presence of LIPI-1 and LIPI-2. PMSCs in the *inlA* gene may lead to the attenuation of virulence, as it may decrease the ability of *L. monocytogenes* to attach to human host cells. In this study, only two novel PMSCs were detected in *inlA* sequences from *L. monocytogenes* strains. This contrasts with the results of previous studies, which have reported that virulence-attenuating mutations in *inlA* are common among *L. monocytogenes* isolates derived from foods, ready-to-eat food processing plants, and retail environments (Nightingale et al., 2008; Van Stelten et al., 2016). This difference in the prevalence of PMSCs in *inlA* may be associated with regional disparities.

The *llsX* (LIPI-3) and *ptsA* (LIPI-4) genes are also considered important virulence factors in *L. monocytogenes*. The LLS protein results in the production of a hemolytic and cytotoxic factor, which contributes to the virulence of the pathogen as determined by murine and human polymorphonuclear neutrophil-based studies. However, rather than contributing to tissue injury and virulence in inner host organs as previously reported, Quereda et al. (2017) demonstrated that LLS production is associated with the destruction of target bacteria. In the present study, five isolates carrying *llsX* were identified among serogroup II.2 isolates, all of which belonged to lineage I. The *llsX* gene was absent from all isolates belonging to serogroup I.1 and III. This observation is consistent with the results of a previous study by Cotter et al. (2008), which first reported that LIPI-3^+^ strains corresponded to representatives of some lineage I STs (STs3–10, 13–14, and 17–19). ST224 (2919-1LM) and ST619 (3950-1LM) were also found to carry *llsX* in this study; this result is in agreement with previous studies reported in China and Switzerland (Althaus et al., 2014; Wang et al., 2018). The hypervirulent clone CC4-associated cellobiose-family PTS is encoded by a six-gene cluster and is responsible for human CNS and maternal-neonatal (MN) listeriosis. *ptsA* is one of the six genes in this PTS (Maury et al., 2016). In this study, 53 (29.44%) isolates carried *ptsA*, mainly from CC87 isolates. This result agrees with findings derived from *L. monocytogenes* isolated from cooked foods and listeriosis cases in China (Wang et al., 2018). These results demonstrated that ST87 strains also carry LIPI-4, which were inconsistent with the results of LIPI-4 specific for CC4 strains reported by Maury et al. (2016). In addition, none of isolates belonging to serogroup I and III were found to carry the *ptsA* gene. While CC8 isolates are also known to cause listeriosis in both China and Switzerland (Althaus et al., 2014; Wang et al., 2018), a comprehensive study is needed to elucidate the pathogenicity of CC8.

Unlike in Western countries where CC1, CC6, CC2, and CC4 are predominant among listeriosis cases (Maury et al., 2016), CC87 is the most frequent ST among listeriosis cases in both mainland China and Taiwan (Huang et al., 2015; Wang et al., 2015, 2018), indicating ST87 predominant in mushroom products may pose a potential of infection threaten to Chinese consumers. Thus, taken together, 98.89% of isolates carried the full-length *inlA*, 29.44% carried *ptsA*, and five carried both *llsX* and *ptsA*; notably, two isolates harbored all three major virulence factors, indicating that these two potential hypervirulent isolates are of considerable public health concern to consumers in China. It is well known that these factors are strongly associated with the infectious potential of *L. monocytogenes* at the population level (Cotter et al., 2008; Maury et al., 2016; Quereda et al., 2016, 2017). These results therefore demonstrate that consumers may be exposed to potential hypervirulent *L. monocytogenes* present in edible mushrooms in China.

## Conclusion

In summary, 21.20% (141/665) of edible mushrooms from the Chinese market system were positive for *Listeria monocytogenes*, while 57.45% (81/141) of these positive samples contained contamination levels of less than 10 MPN/g. Serotypes I.1 (ST8) and II.2 (ST87) were predominant among edible mushrooms, indicating that specific STs may have distinct ecological niches. Most *L. monocytogenes* isolates were resistant to penicillin, ampicillin, oxacillin, and clindamycin; 53.89% exhibited multidrug resistance, while over 90.00% of isolates were susceptible to 16 antibiotics. Virulence profiles showed that potential hypervirulent isolates were found in *L. monocytogenes-*contaminated edible mushrooms, posing a potential public health threat to consumers in China. These findings indicate that the continuous monitoring of the contamination levels of *L. monocytogenes* in edible mushrooms and the exploration of novel control approaches are necessary to ensure the microbiological safety of edible mushrooms.

## Author Contributions

QW, JZ, and MC conceived and designed the experiments. MC, JC, and YC performed the experiments. HZ, QY, and SW conducted bioinformatics analyses. MC, QW, and JZ drafted the manuscript. QW, YD, JW, and SC reviewed the final manuscript. All authors read and approved the final manuscript.

## Conflict of Interest Statement

The authors declare that the research was conducted in the absence of any commercial or financial relationships that could be construed as a potential conflict of interest.

## Footnotes

1 http://bigsdb.pasteur.fr/listeria/listeria.html
