# Supplementary material for: Prevalence, Potential Virulence, and Genetic Diversity of Listeria monocytogenes Isolates From Edible Mushrooms in Chinese Markets
Source: Front Microbiol. 2018 Jul 27;9:1711. doi: 10.3389/fmicb.2018.01711 (PMC6072871; doi:10.3389/fmicb.2018.01711)
Supplement: Supplementary file 1 [file Table_1.DOCX]

**Supplementary Materials**

Table S1 The *Listeria monocytogenes* isolates used in this study

| No. | Isolates | No. | Isolates | No. | Isolates | No. | Isolates |
| --- | --- | --- | --- | --- | --- | --- | --- |
| 1 | 27-1LM | 46 | 749-1LM | 91 | 1646-3LM | 136 | 2600-1LM |
| 2 | 91/0.1LM | 47 | 798-1LM | 92 | 1647-1LM | 137 | 2619-1LM |
| 3 | 132-1LM | 48 | 819-1LM | 93 | 1696-1LM | 138 | 2619-2LM |
| 4 | 180-1LM | 49 | 819-2LM | 94 | 1746-1LM | 139 | 2669-1LM |
| 5 | 243-2LM | 50 | 819-4LM | 95 | 1747-1LM | 140 | 2700-1LM |
| 6 | 268-1LM | 51 | 834-1LM | 96 | 1769-1LM | 141 | 2746-1LM |
| 7 | 268-2LM | 52 | 849-1LM | 97 | 1770-1LM | 142 | 2747-1LM |
| 8 | 283-4LM | 53 | 869-1LM | 98 | 1799-1LM | 143 | 2769-1LM |
| 9 | 283-6LM | 54 | 869-2LM | 99 | 1800-1LM | 144 | 2796-1LM |
| 10 | 298-1LM | 55 | 884-1LM | 100 | 1819-1LM | 145 | 2846-1LM |
| 11 | 318-1LM | 56 | 899-1LM | 101 | 1846-1LM | 146 | 2846-3LM |
| 12 | 318-2LM | 57 | 919-1LM | 102 | 1846-2LM | 147 | 2869-1LM |
| 13 | 348-1LM | 58 | 919-4LM | 103 | 1847-1LM | 148 | 2869-2LM |
| 14 | 348-2LM | 59 | 1069-1LM | 104 | 1869-1LM | 149 | 2871-1LM |
| 15 | 368-1LM | 60 | 1134-1LM | 105 | 1920-1LM | 150 | 2896-2LM |
| 16 | 383-1LM | 61 | 1134-2LM | 106 | 1946-1LM | 151 | 2897-1LM |
| 17 | 385-1LM | 62 | 1169-1LM | 107 | 1947-1LM | 152 | 2897-3LM |
| 18 | 398-1LM | 63 | 1184-1LM | 108 | 1949-1LM | 153 | 2919-1LM |
| 19 | 398-2LM | 64 | 1199-1LM | 109 | 1969-1LM | 154 | 2921-1LM |
| 20 | 418-1LM | 65 | 1219-1LM | 110 | 1969-2LM | 155 | 2947-2LM |
| 21 | 418-2LM | 66 | 1234-1LM | 111 | 1996-1LM | 156 | 3246-1LM |
| 22 | 448-1LM | 67 | 1249-1LM | 112 | 1997-1LM | 157 | 3246-2LM |
| 23 | 448-3LM | 68 | 1269-1LM | 113 | 1997-2LM | 158 | 3296-1LM |
| 24 | 468-1LM | 69 | 1269-4LM | 114 | 1999-1LM | 159 | 3297-1LM |
| 25 | 483-1LM | 70 | 1299-1LM | 115 | 2021-1LM | 160 | 3319-2LM |
| 26 | 518-1LM | 71 | 1349-1LM | 116 | 2035-1LM | 161 | 3346-1LM |
| 27 | 518-2LM | 72 | 1384-1LM | 117 | 2049-1LM | 162 | 3346-3LM |
| 28 | 519-1LM | 73 | 1384-2LM | 118 | 2049-2LM | 163 | 3419-1LM |
| 29 | 548-1LM | 74 | 1386-1LM | 119 | 2069-1LM | 164 | 3546-1LM |
| 30 | 548-3LM | 75 | 1419-1LM | 120 | 2069-2LM | 165 | 3546-2LM |
| 31 | 570-1LM | 76 | 1446-1LM | 121 | 2096-1LM | 166 | 3597-1LM |
| 32 | 598-1LM | 77 | 1447-1LM | 122 | 2169-1LM | 167 | 3671-1LM |
| 33 | 618-1LM | 78 | 1469-1LM | 123 | 2184-2LM | 168 | 3671-2LM |
| 34 | 618-2LM | 79 | 1496-1LM | 124 | 2199-2LM | 169 | 3697-1LM |
| 35 | 633-1LM | 80 | 1519-1LM | 125 | 2246-1LM | 170 | 3697-2LM |
| 36 | 633-2LM | 81 | 1519-3LM | 126 | 2269-1LM | 171 | 3746-1LM |
| 37 | 648-1LM | 82 | 1520-1LM | 127 | 2319-1LM | 172 | 3797-3LM |
| 38 | 648-2LM | 83 | 1547-1LM | 128 | 2319-3LM | 173 | 3870-2LM |
| 39 | 668-1LM | 84 | 1569-1LM | 129 | 2369-1LM | 174 | 3896-1LM |
| 40 | 685-1LM | 85 | 1596-1LM | 130 | 2397-1LM | 175 | 3919-1LM |
| 41 | 698-1LM | 86 | 1597-1LM | 131 | 2420-1LM | 176 | 3949-1LM |
| 42 | 698-4LM | 87 | 1619-1LM | 132 | 2450-1LM | 177 | 3950-1LM |
| 43 | 718-1LM | 88 | 1619-2LM | 133 | 2520-1LM | 178 | 3971-1LM |
| 44 | 733-1LM | 89 | 1646-1LM | 134 | 2546-1LM | 179 | 4150-1LM |
| 45 | 748-1LM | 90 | 1646-2LM | 135 | 2569-1LM | 180 | 4170-1LM |

Table S2 The primers used for serogroups identification and hypervirulent determination of *Listeria monocytogenes* strains

| PCR tests | Target gene | Forward and reverse primers (5'→3') | Specificity | Annealing temperature (°C) | Size of PCR amplicon (bp) | Reference |
| --- | --- | --- | --- | --- | --- | --- |
| Serovar typing | *lmo0737* | AGGGCTTCAAGGACTTACCC | *L. monocytogenes* serovars 1/2a, 3a, 1/2c and 3c | 53 | 691 | Doumith et al. ,2004 |
|  |  | ACGATTTCTGCTTGCCATTC |  |  |  |  |
|  | *lmo1118* | AGGGGTCTTAAATCCTGGAA | *L. monocytogenes* serovars 1/2c and 3c | 53 | 906 | Doumith et al., 2004 |
|  |  | CGGCTTGTTCGGCATACTTA |  |  |  |  |
|  | *ORF2819* | AGCAAAATGCCAAAACTCGT | *L. monocytogenes* serovars 1/2b, 3b, 4b, 4d, 4e and 7 | 53 | 471 | Doumith et al., 2004 |
|  |  | CATCACTAAAGCCTCCCATTG |  |  |  |  |
|  | *ORF2110* | AGTGGACAATTGATTGGTGAA | *L. monocytogenes* serovars 4b, 4d and 4e | 53 | 597 | Doumith et al.,2004 |
|  |  | CATCCATCCCTTACTTTGGAC |  |  |  |  |
|  | *prs* | GCTGAAGAGATTGCGAAAGAAG | All *Listeria* species | 53 | 370 | Doumith et al.,2004 |
|  |  | CAAAGAAACCTTGGATTTGCGG |  |  |  |  |
|  |  | TTTATCCGTACTGAAATTCC |  |  |  |  |
| Hypervirulent determination | *Pts4* | TCCTTTTTCTTTGTTGCGGA | LIPI-4 | 52 | 450 | Maury et al., 2016 |
|  |  | TCTGAAGCTGTACGAAGACA |  |  |  |  |
|  | *inlA* | CGGATGCAGGAGAAAATCC | All *L. monocytogenes* | 55 | 2403 | Wu et al., 2016 |
|  |  | CTTTCACACTATCCTCTCC |  |  |  |  |
|  | *llsX* | TTATTGCATCAATTGTTCTAGGG | LIPI-3 | 52 | 200 | Clayton et al., 2011 |
|  |  | CCCCTATAAACATCATGCTAGTG |  |  |  |  |

Table S3 The primers used for MLST analysis

| Primer | Sequences(5’→3’) | Length (bp) | Tm (°C) |
| --- | --- | --- | --- |
| *abcZoF* | **GTTTTCCCAGTCACGACGTTGTA**TCGCTGCTGCCACTTTTATCCA | 537 | 52 |
| *abcZoR* | **TTGTGAGCGGATAACAATTT**CTCAAGGTCGCCGTTTAGAG |  |  |
| *bglAoF* | **GTTTTCCCAGTCACGACGTTGTA**GCCGACTTTTTATGGGGTGGAG | 399 | 45 |
| *bglAoR* | **TTGTGAGCGGATAACAATTT**CCGATTAAATACGGTGCGGACATA |  |  |
| *catoF* | **GTTTTCCCAGTCACGACGTTGTA**ATTGGCGCATTTTGATAGAGA | 486 | 52 |
| *catoR* | **TTGTGAGCGGATAACAATTT**CAGATTGACGATTCCTGCTTTTG |  |  |
| *dapEoF* | **GTTTTCCCAGTCACGACGTTGTA**CGACTAATGGGCATGAAGAACAAG | 462 | 52 |
| *dapEoR* | **TTGTGAGCGGATAACAATTT**CATCGAACTATGGGCATTTTTACC |  |  |
| *datoF* | **GTTTTCCCAGTCACGACGTTGTA**GAAAGAGAAGATGCCACAGTTGA | 471 | 52 |
| *datoR* | **TTGTGAGCGGATAACAATTT**CTGCGTCCATAATACACCATCTTT |  |  |
| *ldhoF* | **GTTTTCCCAGTCACGACGTTGTA**GTATGATTGACATAGATAAAGA | 453 | 52 |
| *ldhoR* | **TTGTGAGCGGATAACAATTTC**TATAAATGTCGTTCATACCAT |  |  |
| *lhkAoF* | **GTTTTCCCAGTCACGACGTTGTA**AGAATGCCAACGACGAAACC | 480 | 52 |
| *lhkAoR* | **TTGTGAGCGGATAACAATTT**CTGGGAAACATCAGCAATAAAC |  |  |
| *LhkA*-F3 | GCAAGTTTTGAATACGTATCAGCG (Lineage 3) | 480 | 52 |
| *LhkA*-R2 | TACGCATTTCATGAGAAACATCAG (Lineage 3) |  |  |
